# Real-Time Anomaly Detection for an ADMM-Based Optimal Transmission Frequency Management System for IoT Devices

**DOI:** 10.3390/s22165945

**Published:** 2022-08-09

**Authors:** Hongde Wu, Noel E. O’Connor, Jennifer Bruton, Amy Hall, Mingming Liu

**Affiliations:** 1 School of Electronic Engineering, Dublin City University, Dublin 9, Ireland; 2 Insight SFI Research Centre for Data Analytics, Dublin City University, Dublin 9, Ireland

**Keywords:** anomaly detection, Internet of Things, decentralised algorithms, edge intelligence

## Abstract

In this paper, we investigate different scenarios of anomaly detection on decentralised Internet of Things (IoT) applications. Specifically, an anomaly detector is devised to detect different types of anomalies for an IoT data management system, based on the decentralised alternating direction method of multipliers (ADMM), which was proposed in our previous work. The anomaly detector only requires limited information from the IoT system, and can be operated using both a mathematical-rule-based approach and the deep learning approach proposed in the paper. Our experimental results show that detection based on mathematical approach is simple to implement, but it also comes with lower detection accuracy (78.88%). In contrast, the deep-learning-enabled approach can easily achieve a higher detection accuracy (96.28%) in the real world working environment.

## 1. Introduction

The Internet of Things (IoT) is a network including smart devices which can connect, exchange and share data with each other over the Internet [1]. An IoT system can capture real-time environmental data through sensors embedded in IoT devices. The data emitted from the system can be transmitted to the cloud via gateways for further storage, process and analysis [2]. Typically, in a cloud-dominant centralised architecture, artificial intelligence (AI)-enabled computing nodes are often integrated and implemented at the cloud side, with an intention to fetch the useful information from the transmitted data centrally and provide better insight for users to make decisions. Some recent IoT applications relying on this architecture are described in [3,4,5]. However, such an IoT system setup is highly dependent on the reliability of a remote cloud instance and often struggles to handle real-time data analysis and inference tasks with low latency requirements, e.g., anomaly detection. As a result, deploying AI on the edge, i.e., edge AI, becomes more important for these tasks. Moreover, edge AI provides an effective way for local edge devices to be engaged in such activities with better privacy awareness and enhanced system reliability, especially when a cloud instance could be delayed or fail in response in practical scenarios [6,7,8,9].

Our objective in this work is to further explore the benefits of edge AI. Specifically, we wish to investigate how edge AI can be leveraged to carry out real-time anomaly detection in the IoT system proposed in our previous work [10], where a group of IoT devices work collaboratively to seek for an optimal resource allocation scheme of their transmission frequencies using the alternating direction method of multipliers (ADMM)-based optimisation method. We note that each IoT edge device in the proposed system [10] has the potential to adjust its allocated data transmission frequency through a user-defined utility function, which is only accessible to each local device, and some model parameters. In a real-world setup, this implies that a cyberattacker may wish to manipulate the functions in order to implicitly increase or decrease the transmission frequency of any IoT devices for his own benefits in attacking the network. For instance, an external attacker may sneak into the network to inject malicious codes into the key process of the application in order to effectively control the data transmission frequency of the tampered devices. To further illustrate this point, for example, an external attacker may hack into a critical camera facilitated in the bank system to reduce the number of frames from the camera transmitted to the cloud for remote monitoring, clearly leading to an anomalous and dangerous behaviour for the whole IoT system.

Given this context, the purpose of this work is to design an anomaly detector which is able to find out any local device that *implicitly* tampers its transmission frequency by modifying its utility function and/or its model parameters in real time. More specifically, we wish to set up a mechanism at the gateway level to timely identify anomalies within the IoT network, where no user-defined information for any local IoT device needs to be explicitly exposed to the detector for anomaly analysis. The overarching goal of the anomaly detector is to ensure the underlying IoT system can be operated in a fair and proper manner without sacrificing users’ privacy, i.e., the user-defined information. It is important to note that we do not focus on the detection of anomalies in *data contents*, e.g., changing a data entry in the transmitted json file from value “A” to “B” [11]; instead, we are interested in detecting the change of transmission pattern as a result of the anomalies happening on the edge devices as aforementioned. With this in mind, our contributions to this work can be summarised as follows:

1. The key focus of our previous work [10] was to propose an optimisation framework for an IoT network so that the transmission frequency of the connected IoT devices can be dynamically adjusted to their optimal values through an ADMM-based iterative optimisation method. In this work, we focus on the design of an anomaly detector on top of the system we proposed in [10], which is able to infer anomalies that may occur in the underlying IoT transmission management system in real time. Thus, the scope of work is significantly extended in comparison to [10].
2. We propose both mathematical-rule-based and deep-learning-based approaches for detecting anomalies in the IoT transmission frequency management system. In particular, the rule-based approach is designed to reveal anomalies of the system based on fundamental optimisation theory, and the deep learning approach aims to establish a prediction model based on sequential data analysis in system implementations.
3. We conduct a comprehensive comparative study using both anomaly detector strategies and demonstrate the strengths and weaknesses for the two approaches in both simulated and practical working environments.

The remainder of this paper is organised as follows. Related works are presented in Section 2, where we mainly review the previously proposed IoT data transmission frequency management system and its links to the anomaly detection problem. The anomaly detection problem is then solidly formulated in Section 3, with two proposed solutions discussed in Section 4. Experimental setups are illustrated in Section 5 and results are discussed in Section 6. Finally, discussion and conclusion for the current research and potential future research directions are presented in Section 7 and Section 8, respectively.

## 2. Related Works

We first review the optimal transmission frequency management system in our previous work [10] followed by a review of related works in relation to anomaly detection for IoT.

### 2.1. Transmission Frequency Management System

**Figure 1:**
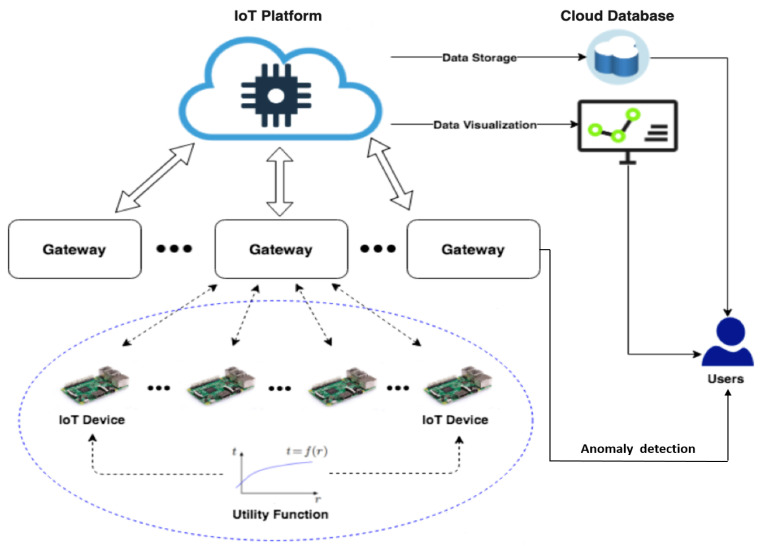
Schematic diagram of the system architecture [10].

We considered a data transmission frequency management system in [10], the schematic diagram of which is shown in Figure 1. The system consists of edge devices, gateways, a remote IoT platform, and a cloud database with users, and it operates as follows. At the beginning, a group of IoT devices communicates with the gateway for decentralised optimisation. During the optimisation process, transmission frequencies are calculated iteratively with respect to the system resource constraints specified at the gateway. Once transmission frequencies are converged and allocated locally, the devices then push data streams to the cloud database as per the obtained optimal transmission frequency through the gateway and the IoT platform. Users can visualise data flows using specific applications at the cloud side.

Mathematically, we considered the following problem formulation:

(1) $$\begin{array}{c} \max_{x_{1},x_{2},\ldots ,x_{N}}\;\sum_{i=1}^{N}h_{i}\left(x_{i}\right),\\ \mathrm{such}\;\mathrm{that}\;\sum_{i=1}^{N}x_{i}\leq c,\;\sum_{i=1}^{N}a_{i}x_{i}\leq d,\;x_{i}\geq 0 \end{array}$$

where *c* is defined as the maximum writing frequency (MWF) to a database, *d* is the total available storage capacity per given time slot, $a_{i}$ is the requested data size for transmission per given time slot specified by the device *i*, and $h_{i}\left(x_{i}\right)$ is the utility function defined by the *i*th device for a given data flow writing frequency (DFWF) $x_{i}$. In this setup, both *c* and *d* are limited resources, and a group of *N* devices intend to find out their optimal $x_{i}$ by solving the optimisation problem (1) in a cooperative manner. Clearly, an optimal solution for any device in the network depends on both user-defined information, i.e., $h_{i}$ and $a_{i}$, as well as other system-level parameters involved, i.e., *N*, *c*, and *d*.

**Comment:** A utility function $h_{i}$ essentially captures how a device *i* can practically benefit from a given DFWF, i.e., the more DFWF is allocated, the better the quality of experience (QoE)/utility [12,13] value that can be achieved. In particular, we assumed that each utility function $h_{i}$ is concave, which is a common assumption in the literature, e.g., [14,15], implying a diminishing marginal utility in using the system. In this context, a change of utility function for a specific device may also imply the change of status of the device [16]. For example, a carbon monoxide monitoring IoT device may be associated with *multiple user-defined utility functions* reflecting the latest condition of the device, triggered by various events. In a fire alarm scenario, the utility function of the device can be defined to switch to a different state, permitting the monitoring device to access greater system resources and allowing for additional details from the scene to be included in the data transmission. For example, the carbon monoxide density data were transmitted once per minute in the normal situation, but now they need to be transmitted once per second in this emergency scenario. Thus, it is important to keep this information local and prevent malicious tampering.

For optimisation, the decentralised ADMM algorithm [17] is applied and is implemented following Algorithm 1. The idea of applying a decentralised algorithm is that each utility function is separately defined on the edge device, so that parameter $x_{i}$ and $u_{i}$ can update in parallel *without revealing the information of $h_{i}$*. Given the system constraints, the projection operator $\Pi_{C}$ converts any input vector to its confined space specified by the linear constraints C. Specifically, the update of z is conducted on the gateway taking the vector $x^{t+1}+u^{t}$ as input, and updates on $x_{i}$ and $u_{i}$ are on local edge devices, with ρ being defined as the augmented Lagrangian parameter.
**Algorithm 1** Decentralised ADMM algorithm.1:${x_{i}}^{t+1}:={\mathrm{argmax}}_{x_{i}}(f_{i}\left(x_{i}\right)+\left(\rho /2\right)\left|\right|{x_{i}}^{t}-{z_{i}}^{t}+{u_{i}}^{t}{\left|\right|}_{2}^{2})$2:$z^{t+1}:=\Pi_{C}\left(x^{t+1}+u^{t}\right)$3:${u_{i}}^{t+1}:={u_{i}}^{t}+{x_{i}}^{t+1}-{z_{i}}^{t+1}$

**Figure 2:**
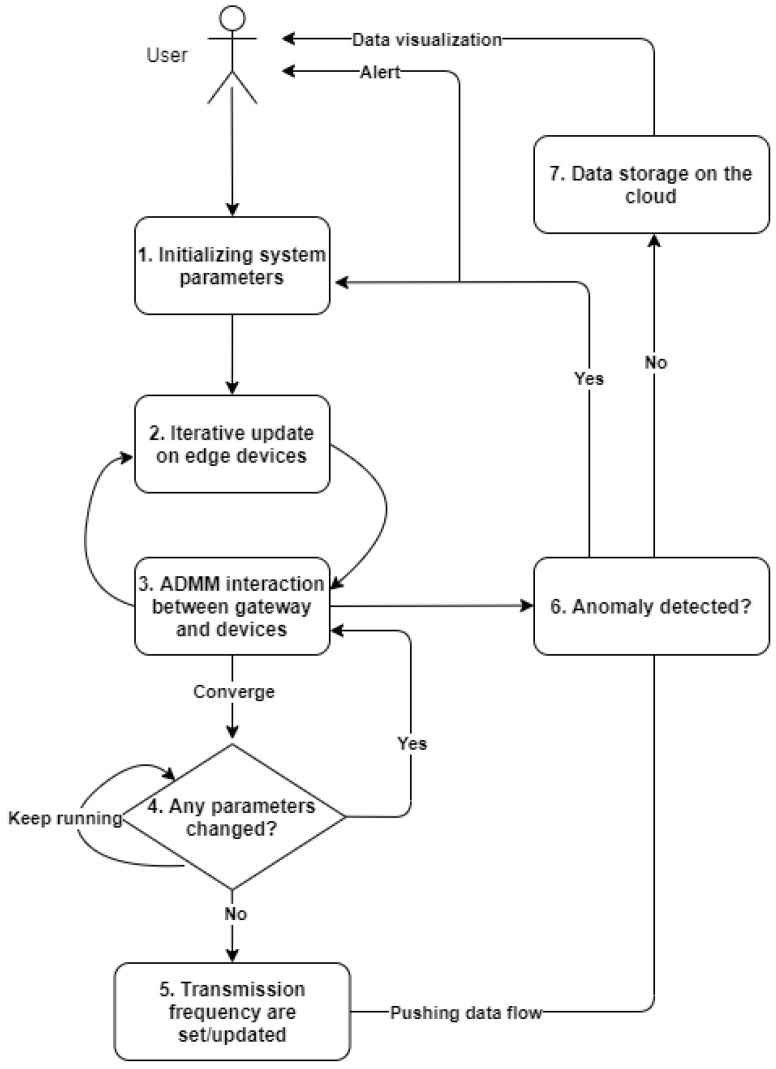
System implementation flowchart [10].

The flowchart for the whole system implementation is presented in Figure 2. The role of an anomaly detector is described in Step 6, where, in real time, it alerts the user of an anomaly and resets the system to its normal state.

### 2.2. Related Solution for Anomaly Detection

Referring to the works in the literature, anomalies for IoT can be broadly classified into one of the following three types [18,19]:

- Point: Anomalies happen randomly without clear reason and always with irregularity. For instance, network sensors can catch a sudden fluctuation in video signals [20] due to abnormal noises.
- Contextual: Anomalies happen given the specific context, including the spatial and temporal cues. For example, in [21], the pattern related to traffic accident varies between long-term and short-term prediction (e.g., day-level and hour-level prediction) in different areas.
- Collective: Anomalies happen when the central node and edge devices work incongruously or the observation in a group has unusual patterns with other groups. For instance, in [22], anomalies can be defined as cascading delays in railway traffic, and these delays are common in traffic data across different weeks.

Our work focuses on detection of contextual anomalies, considering that anomalies happen in the specific IoT context and the gateway keeps monitoring the system parameters both before and after an anomaly occurs (i.e., temporal cues). We note that significant work has been undertaken in this area: for instance, Liu et al. [23] proposed a detector for on and off attack by a malicious network node in an industrial IoT site; Anthi et al. [24] represented an intrusion detection system for an IoT system to identify the denial of service (DoS) attacks; Ukil et al. [25] discussed the detection of anomalies in healthcare analytics based on IoT by analysing the cardiac signal; and Hu et al. [26] proposed a context-augmented graph auto-encoder (Con-GAE) for anomaly detection in traffic monitoring. However, the anomalies defined in these works are largely based on *tempering contents in data packets* transmitted by IoT devices, and no approach has been found for anomaly detection for an IoT data transmission frequency system involved with an optimal iterative scheme. Moreover, a fundamental difference between our definition and theirs is that we focus on detecting the anomalies caused by the abnormal transmission pattern of IoT devices in the network, which has no regard to the contents in the transmitted data packets.

## 3. Problem Statement

Given the transmission frequency management system described in Figure 1 and problem (1), manipulations on the edge (i.e., including edge devices and gateway) can lead to changes in transmission frequencies. From a threat modelling perspective, for instance, with reference to the MITRE framework [27], adversaries may manipulate the transmission frequency of a given IoT device through external remote access, and maliciously inject codes through process injection to eventually escalate the privilege of the device for specific purposes. Specifically, in this work we assume that such manipulations can happen by changing the utility function input, function type, data size request per writing request (i.e., defined on edge devices), maximum writing frequency, and data storage (i.e., system resource allocated to the gateway). In general, when manipulations happen on the device *j* in the network, a new optimisation process needs to be reactivated by solving the following problem:

(2) $$\begin{array}{c} \max_{x_{1},x_{2},\ldots ,x_{N}}\;\sum_{i=1,i\neq j}^{N}h_{i}\left(x_{i}\right)+h_{j}^{*}\left(x_{j}\right),\\ \mathrm{subject}\;\mathrm{to}\;\sum_{i=1}^{N}x_{i}\leq c,\sum_{i=1,i\neq j}^{N}a_{i}x_{i}+a_{j}^{*}x_{j}\leq d,\;x_{i}\geq 0 \end{array}$$

where $h_{j}^{*}$ and $a_{j}^{*}$ denote the new utility function and new data packet size after tampering, respectively.

Clearly, there are many ways that an optimal transmission frequency $x_{j}$ can be implicitly tampered. In our context, we consider the following specific definitions:

1. **Manipulation on utility function input only**: The independent variable of the utility function is manipulated by adding an input factor with a small given range, $h_{j}\left(x_{j}\right)\Rightarrow h_{j}\left(x_{j}+\mathrm{input}\;\mathrm{factor}\right)$.
2. **Manipulation on utility function type and input**: The utility function can be totally changed to anther type of concave function specified by the utility function set of the system, i.e., $h_{j}\left(x_{j}\right)\Rightarrow h_{j}^{*}\left(x_{j}+\mathrm{input}\;\mathrm{factor}\right)$. Note that this setting maps to the “multiple user-defined utility functions” example in Section 2.1.
3. **Manipulation on transmission data size**: The data size $a_{j}$ required for the *j*’th device per writing request is manipulated by adding a size factor with a small given range, $a_{j}\Rightarrow a_{j}+\mathrm{size}\;\mathrm{factor}$.

**Comment:** It is also possible to affect the optimal transmission frequency $x_{j}$ and $x_{i},i\neq j$ by manipulating system resource in a small given range, such as c⇒c+MWF factor and d⇒d+storage factor. In our definition, such manipulations are regarded as systematic adjustment as it is not directly related to any user-specific property, e.g., $h_{j}$, and thus it will be regarded as normal scenarios in our anomaly detection analysis.

In addition, we also have the following assumptions in our problem statement.

**Figure 3:**
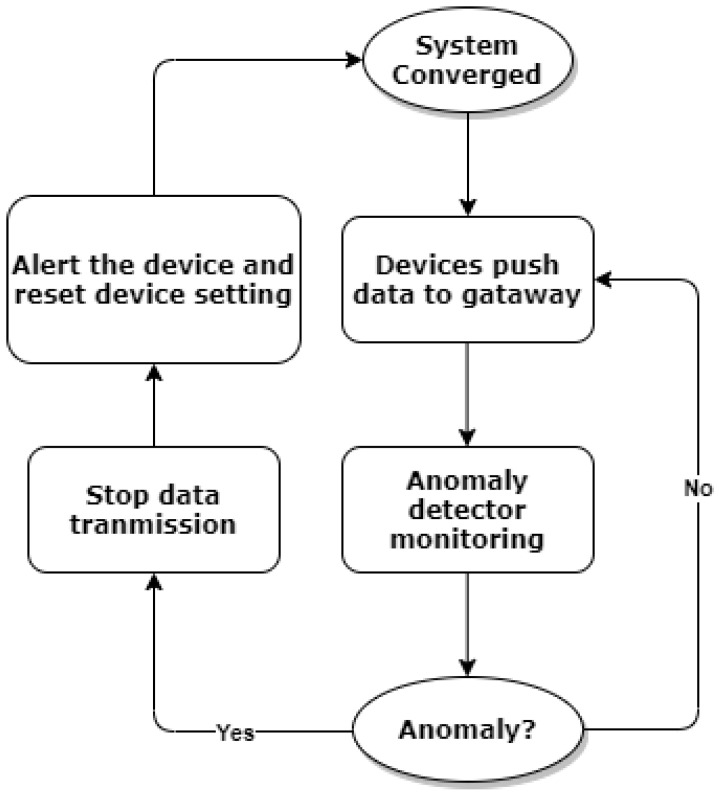
Anomaly detection and response process.

1. We assume that at every given time only one edge device is manipulated, which is the fundamental basis for detecting an anomaly when multiple devices are manipulated in our system.
2. We assume that the anomaly detector is a separate process running on the gateway, and it can only access limited information on the gateway but not all. More specifically, we assume that the anomaly detector can only access the value of z and the sum of x and u, denoted by v, from the ADMM iterative process at the gateway. It will never access the exact transmission frequency x directly from the local devices and other resources/parameters shared between devices and the gateway.
3. We assume that the anomaly detector starts to monitor anomalies in real time once the ADMM algorithm converges and local devices start pushing data to the gateway. The device setting will be reset when any anomalies are detected, and the optimisation process will be reactivated to reset the optimal solutions for fair resource allocation as per the normal situation. To further illustrate this point, the process of anomaly detection is shown in Figure 3.

## 4. Proposed Approach

We now introduce two approaches to address the anomaly detection problem defined in Section 3, namely, a rule-based approach and a deep learning approach. The rule-based approach detects system anomaly based on the mathematical deduction, and the deep learning approach solves the detection problem using collected experimental datasets of the system. The rule-based approach is proposed as a baseline method; as we shall see, it has some drawbacks in detecting system anomalies in details.

### 4.1. Rule-Based Anomaly Detection

Our objective is to investigate the behaviours of the optimised system before and after a manipulation. To this end, we borrow some fundamental concepts from the optimisation theory, i.e., the Karush–Kuhn–Tucker (KKT) conditions [28], for the optimisation problem (1) under study. For mathematical conventions, we now rewrite the original optimisation problem (1) in the following format:

(3) $$\begin{array}{c} \min_{x_{1},x_{2},\cdots ,x_{N}}\;\sum_{i=1}^{N}f_{i}\left(x_{i}\right),\\ s.t.\;\sum_{i=1}^{N}x_{i}\leq c,\;\sum_{i=1}^{N}a_{i}x_{i}\leq d,\;x_{i}\geq 0 \end{array}$$

where $f_{i}\left(x_{i}\right):=-h_{i}\left(x_{i}\right)$ is a convex function. The Lagrange equation of (3) is presented as follows:

(4) $$\begin{array}{c} L\left(x,\lambda_{1},\lambda_{2}\right)=\sum_{i=1}^{N}f_{i}\left(x_{i}\right)+\lambda_{1}g_{1}\left(x\right)+\lambda_{2}g_{2}\left(x\right) \end{array}$$

and the KKT conditions require the following to be held for optimality:

(5) $$\begin{array}{c} \frac{\partial L}{\partial x_{i}}=\frac{\partial f_{i}\left(x_{i}\right)}{\partial x_{i}}+\lambda_{1}\frac{\partial g_{1}\left(x\right)}{\partial x_{i}}+\lambda_{2}\frac{\partial g_{2}\left(x\right)}{\partial x_{i}}=0,\\ \lambda_{1},\lambda_{2}\geq 0,\\ \lambda_{1}g_{1}\left(x\right),\lambda_{2}g_{2}\left(x\right)=0 \end{array}$$

where *∂* is the operation of partial derivative (i.e., gradient), and $\lambda_{1}$, $\lambda_{2}$ are Lagrange coefficients for $g_{1}\left(x\right)$, $g_{2}\left(x\right)$, and $x=(x_{1},x_{2},\cdots ,x_{N})$.

Specifically, $g_{1}\left(x\right)=\sum_{i=1}^{N}x_{i}-c$ and $g_{2}\left(x\right)=\sum_{i=1}^{N}a_{i}x_{i}-d$, which represents the constraints in problem (3) with

(6) $$\begin{array}{c} \frac{\partial g_{1}\left(x\right)}{\partial x_{i}}=1,\\ \frac{\partial g_{2}\left(x\right)}{\partial x_{i}}=a_{i}. \end{array}$$

Clearly, the converged optimal solution will fall into one of the following situations with reference to system constraints.

#### 4.1.1. Situation $g_{1}\left(x\right)=0$, $g_{2}\left(x\right)<0$

Given $g_{2}\left(x\right)<0$, we have $\lambda_{2}=0$ according to Equation (5). The system is running under $\sum_{i=1}^{N}x_{i}=c$. Thus, for each device *i*, we have

(7) $$\begin{array}{c} \frac{\partial L}{\partial x_{i}}=\frac{\partial f_{i}\left(x_{i}\right)}{\partial x_{i}}+\lambda_{1}\frac{\partial g_{1}\left(x\right)}{\partial x_{i}}\\ =\frac{\partial f_{i}\left(x_{i}\right)}{\partial x_{i}}+\lambda_{1}=0, \end{array}$$

That is

(8) $$\begin{array}{c} \frac{\partial f_{1}\left(x_{1}\right)}{\partial x_{1}}=\frac{\partial f_{2}\left(x_{2}\right)}{\partial x_{2}}=\cdots =\frac{\partial f_{N}\left(x_{N}\right)}{\partial x_{N}} \end{array}$$

for problem (3).

Considering the constraint $\sum_{i=1}^{N}x_{i}=c$, when a manipulation results in an increase of DFWF for device *j* (i.e., $x_{j}$), at least one of $x_{i}$ (i≠j) decreases. Considering that $\frac{\partial f_{i}\left(x_{i}\right)}{\partial x_{i}}$ is monotonously increasing with respect to an increased $x_{i}$ (i.e., with convexity), the decrease of $x_{i}$ will also decrease $\frac{\partial f_{i}\left(x_{i}\right)}{\partial x_{i}}$. Consequently, $\frac{\partial f_{i}\left(x_{i}\right)}{\partial x_{i}},\forall i\neq j$, will decrease as per (8), which indicates decrease of $x_{i},\forall i\neq j$. Therefore, an increase of $x_{j}$ results in the decrease of transmission frequencies of all other devices $x_{i},\forall i\neq j$.

#### 4.1.2. Situation $g_{1}\left(x\right)<0$, $g_{2}\left(x\right)=0$

Given $g_{1}\left(x\right)<0$, we have $\lambda_{1}=0$ according to Equation (5). The system is running under $\sum_{i=1}^{N}a_{i}x_{i}=d$. For each *i*, we have

(9) $$\begin{array}{c} \frac{\partial L}{\partial x_{i}}=\frac{\partial f_{i}\left(x_{i}\right)}{\partial x_{i}}+\lambda_{2}\frac{\partial g_{2}\left(x\right)}{\partial x_{i}}\\ =\frac{\partial f_{i}\left(x_{i}\right)}{\partial x_{i}}+\lambda_{2}a_{i}=0, \end{array}$$

where $a_{i}\geq 0$ since $a_{i}$ is the required data size. This implies

(10) $$\begin{array}{c} \frac{\partial f_{1}\left(x_{1}\right)}{\partial x_{1}}=\frac{a_{1}}{a_{2}}\frac{\partial f_{2}\left(x_{2}\right)}{\partial x_{2}}=\cdots =\frac{a_{1}}{a_{N}}\frac{\partial f_{N}\left(x_{N}\right)}{\partial x_{N}} \end{array}$$

for problem (3).

Similar to the first situation, without loss of generality, an increase of DFWF for device *j*, $x_{j}$, after a manipulation will lead to a decrease of at least one $x_{i},i\neq j$ due to the equality constraint $g_{2}\left(x\right)=0$. Since $f_{i}\left(x_{i}\right)$ is convex, the decrease of $x_{i}$ indicates a decrease of $\frac{\partial f_{i}\left(x_{i}\right)}{\partial x_{i}}$. Given Formula (10), we have that $\frac{\partial f_{i}\left(x_{i}\right)}{\partial x_{i}},\forall i\neq j$ decreases proportionally followed by the increase of $x_{j}$, resulting a reduced $x_{i},\forall i\neq j$.

#### 4.1.3. Situation $g_{1}\left(x\right)<0$, $g_{2}\left(x\right)<0$

Given $g_{1}\left(x\right)<0$ and $g_{2}\left(x\right)<0$, we have $\lambda_{1}=0$ and $\lambda_{2}=0$ according to Equation (5). Thus, the system is running within the boundary of system resources. For each device *i*, we have

(11) $$\begin{array}{c} \frac{\partial L}{\partial x_{i}}=\frac{\partial f_{i}\left(x_{i}\right)}{\partial x_{i}}, \end{array}$$

Considering that the system is running within the boundary of system resources, manipulation on any device will not affect other devices. That is, for instance, when a manipulation results in an increase of DFWF for device *j* (i.e., $x_{j}$), other $x_{i},\forall i\neq j$, remain unchanged since they were already optimised and the system resource is sufficient to cover the extra needs for device *j*.

**Figure 4:**
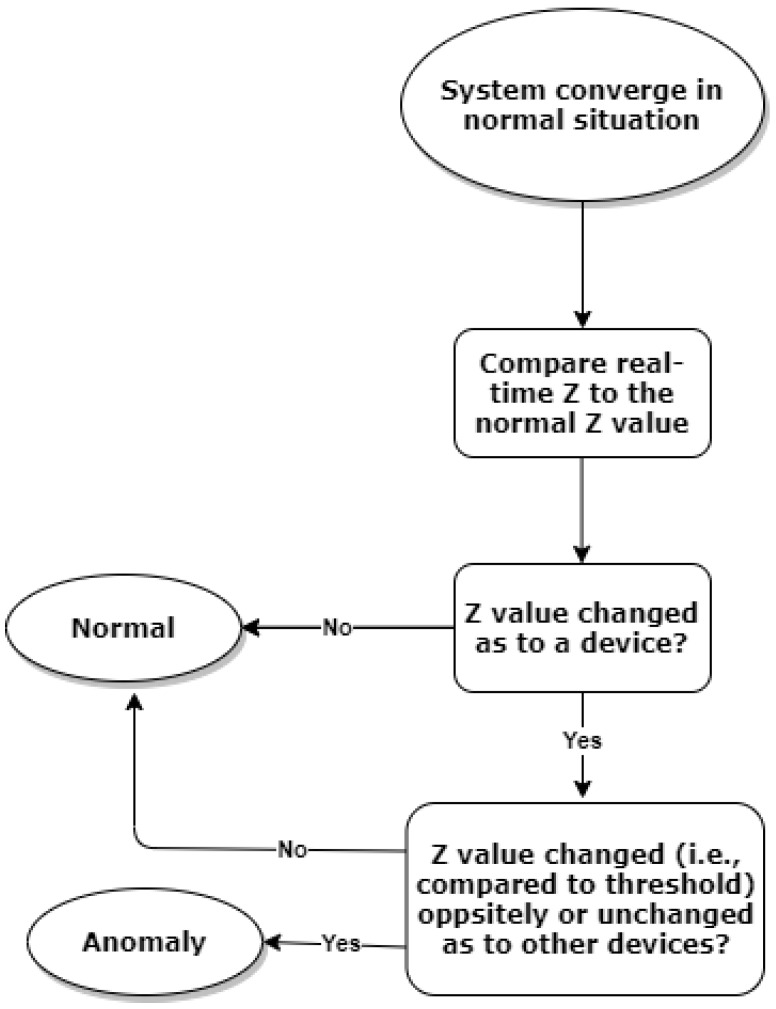
The proposed rule-based detection process.

Given the above discussion, we observed that once the manipulation accounts for a change of DFWF on a given device, DFWF of other devices will either change oppositely or remain unchanged. In the decentralised ADMM Algorithm 1, the parameter z will converge to the value of DFWF (i.e., x) when the optimal DFWF is allocated. Thus, the value of z essentially captures dynamic information in relation to the convergence of DFWF, which means that it can be used to infer the potential anomaly of DFWF of IoT devices at the gateway level. Accordingly, we can devise a simple rule-based mechanism for anomaly detection, and the flow chart is shown in Figure 4. It operates as follows. When the system starts to operate and converges to optimality normally, the anomaly detector keeps a record of the normal z value while keeping monitoring the z value from the algorithm iteration in real time. Once the absolute difference between the observed z value and the normal z value becomes greater than a preset threshold (component-wise), the anomaly for the corresponding device is recorded. In this work, the thresholds are defined as 1%, 5%, 10%, 15%, 30%, and 50% to the change of the recorded normal z value so that the performance of the approach can be evaluated comprehensively.

**Figure 5:**
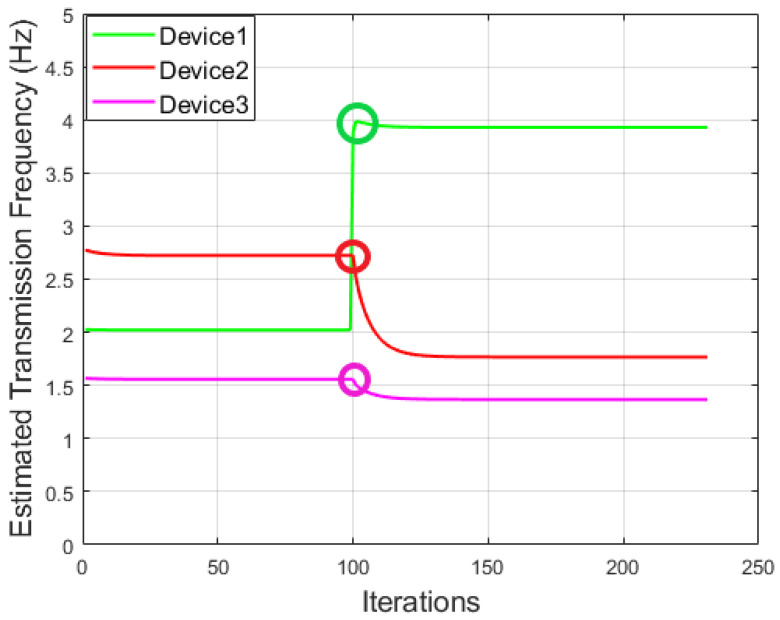
An example to illustrate the change of transmission pattern due to the manipulation of device 1.

To further demonstrate how we can apply the rule-based approach for anomaly detection, a simple simulation is conducted on the IoT system consisting of three devices. The utility functions for all three devices are reported in Table 1, where we assumed that the first device, i.e., device 1, was manipulated by only adding an *input factor* =−2 at a given point during our experiment. Our results are shown in Figure 5. It can be seen that device 1 was manipulated at the 100th iteration, indicated by different cycles highlighted in Figure 5, leading to an increase by 94% (i.e., from 2.02 to 3.93) in DFWF, while device 2 and device 3 reduced their transmission frequencies by 35% and 12% correspondingly. Therefore, by applying a threshold lower than 12% to the change of recorded normal *z* values, the rule-based detector can detect the increase of transmission frequency in device 1 and the decrease of transmission frequencies in device 2 and device 3 successfully. Given this, an anomaly will be spotted in this case.

### 4.2. Limitations on the Rule-Based Anomaly Detection

Our results in Section 4.1 show that a rule-based approach has potential for anomaly detection as long as the manipulation leads to a change of transmission frequency. However, such an approach also has certain limitations when deployed in the real world, which are now summarised as follows:

A. The rule-based approach mainly relies on the optimality criteria without fully leveraging information from the iterative process, and as a result it cannot further distinguish different types of anomalies when a manipulation happens on the edge device.
B. As we shall see, system parameters, i.e., z, may fluctuate during the optimisation process, and this can easily result in misjudgements when using the rule-based approach.
C. Furthermore, when there are network delays in the IoT network, transmission frequencies of the devices may not change simultaneously, which can also lead to misjudgements when using the rule-based approach.

Due to the uncertainty of a practical running IoT environment as well as the depth of information that can be leveraged from the collected data for anomaly detection, we are also interested in exploring a data-driven-based solution to address the limitations exposed by the rule-based approach, which is introduced in the following section.

### 4.3. IoT Anomaly Detection with LSTM-Based Approaches

In this section, deep-learning-based approaches are proposed for anomaly detection on the gateway, covering all categories of the anomalies defined in Section 3. Our starting point is the observation that an anomaly detector can only access the value of z and the sum of x and u, i.e., v, at every given time point of interest, i.e., a sequential data. Inspired by this, we aim to leverage a prevalent sequence-based prediction model, i.e., long short-term memory (LSTM) [29] as our starting point, which is well known for addressing anomaly detection problem in time-series data [30,31].

Specifically, we apply a basic one-layer LSTM architecture in our model design and compare the detection performance with different complicated variants which have been applied in anomaly detection, e.g., bidirectional LSTM (bi-LSTM) [32], stacked-LSTM [33], LSTM with attention mechanism [34] (LSTM-attention), and LSTM with encoder techniques [35] (LSTM-encoder), assuming that extra deep learning layers may help improve the detection accuracy. Let $X_{t}$ denote the input feature at step t (i.e., the *t*th iteration of the ADMM algorithm), then the LSTM network essentially extracts hidden information at each step, *t*, and feeds this in as the input of the next step, t+1. A standard LSTM unit includes a cell, a forget gate, an input gate, and an output gate to jointly manage the information flow from input to output. The input feature $X_{t}$ can be either a scalar, vector, or matrix. In our case, the input feature $X_{t}$ is represented as a matrix consisting of system parameters of each device, *i*, from iteration *t* to t+n. Here, the input features contain $\left[v_{i}^{t},\cdots ,v_{i}^{t+n}\right]$, $\left[z_{i}^{t},\cdots ,z_{i}^{t+n}\right]$, where $v_{i}:=x_{i}+u_{i}$. The output of the LSTM model is the categorical label for the anomaly corresponding to the manipulation types as per our definition.

## 5. Experimental Setup

In this section, we introduce specific settings for manipulations, IoT system implementation, data generation, and the LSTM network in our experiment.

### 5.1. Setup for Manipulations

It is worth noting that figuring out how to define a user’s preference using a utility function is an open issue [36], as different users may end up having totally different utility values with respect to a given source, i.e., DFWF in our case. However, in our context, we shall make the assumption that such a function is concave as it generally reflects the fact that a user’s satisfaction level is increased when the allocated DFWF is also increased. With this in mind, we have the following settings:

- **Manipulation on utility function type and input**: The utility function is changed from $f_{j}\left(x_{j}\right)$ to $f_{j}^{*}\left(x_{j}\right)$ (i.e., see Table 2) with *input factor*, resulting in manipulation $f_{j}\left(x_{j}\right)\Rightarrow f_{j}^{*}(x_{j}+$ *input factor*), labelled as type 1.
- **Manipulation on transmission data size**: The data size factor is set as a random value from the set of [−1,1] and the $a_{j}$ is manipulated as $a_{j}\Rightarrow a_{j}+$ *size factor*, labelled as type 2.
- **Manipulation on utility function input only**: In this case, the inputfactor is set as a random value from the set of [−3,3] for the manipulation $f_{j}\left(x_{j}\right)\Rightarrow f_{j}(x_{j}+$ *input factor*), which is labelled as type 3.

**Comment:** As mentioned in Section 3, manipulating system resources can also affect the optimal transmission frequencies for edge devices, but it will be treated as normal systematic adjustment. Regarding manipulation of system resources, the MWF, *c*, and data storage amount, *d*, are manipulated by adding an MWF factor and storage factor. The factors *c* and *d* are attributed a value from the set of [−3,3], and [−5,5] respectively, ensuring that the manipulated *c* and *d* are positive. Here, we have manipulation c⇒c+ *MWF factor* and d⇒d+ *storage factor* which are labelled as normal (type 0).

### 5.2. System Setup

In general, there are two different system setups in experiments. One simulates the ideal IoT scenario including an arbitrary number of devices, without considering the effects of network delay. The second simulates a practical IoT environment involving real IoT devices with network delay. In order to compare the performance of the two setups, we manually trigger manipulations and record the manipulation count/type for both systems. However, considering that in a real-world environment it is impossible for a gateway to know the ground truth, we deploy the pretrained model based on the ideal scenario and evaluate the performance of the model in the practical IoT environment.

**Figure 6:**
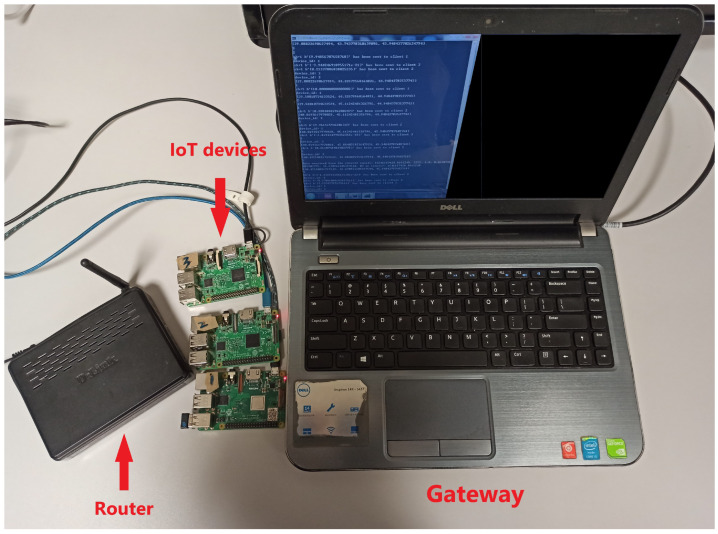
System device setup [10].

More specifically, the simulation system (SS) and real-world system (RS) are introduced to validate the performance of the anomaly detector. The SS simulates the ideal scenario that all devices transmit data to the gateway without network delay. In our experiment, SS includes the virtual edge devices and gateway on a local computer where the data streams can be exchanged even if there is no network environment. The RS simulates the practical IoT application where all devices transmit data to the gateway with network delay effect being considered. In our real-world implementation, this consists of three edge devices (i.e., Raspberry Pis) and a laptop acting as the gateway. Edge devices communicate with the gateway through a wireless router, as shown in Figure 6. The key system properties for this practical system are set as $N=3,c=10,d=20,a_{1}=2,a_{2}=3,$ and $a_{3}=5$.

It is worth noting that in SS we simulate the ideal scenario and generate data for the purpose of training the anomaly detector. Therefore the data are labelled corresponding to anomalies when devices are manipulated. In RS, we simulate the scenario where edge devices are implemented in a real-world IoT network for daily service. In this context, the data collected from RS are without labels and are used for anomaly detection in real-world applications.

### 5.3. Data Generation

The process of data generation can be summarised as follows: during the daily service of the IoT network, the system suffers attacks and transmits a data flow containing unexpected transmission frequencies, and the system returns to its normal state after the attacks end. Specifically, at the beginning, SS and RS are running under the normal state. After the ADMM algorithm has converged for the duration of several ADMM iterations, a type of manipulation happens on the IoT devices and the system reacts, calculating new transmission frequency values. After the anomaly happens and the ADMM algorithm converges under the anomaly, the edge devices return to the normal state and the system repeats the process. Here, we assume that the normal operation period is usually longer than the abnormal period in a real-world IoT application [37]. As our numerical setting, we assume that the duration of normal states can vary between 100 and 120 iterations, and an anomaly can last between 50 and 70 iterations. During this cycle, the normal situation is labelled as type 0 and anomalies are labelled as different numeric types. Data (i.e., ADMM parameter) z and v generated from the ADMM algorithm are recorded along each iteration during the interaction between the gateway and the edge devices. Data are fully labelled as either normal (type 0) or anomalous (type 1, 2, 3) and attributed to either SS or RS. Data generated from SS are called simulation set while those generated from RS are called practical set.

**Figure 7:**
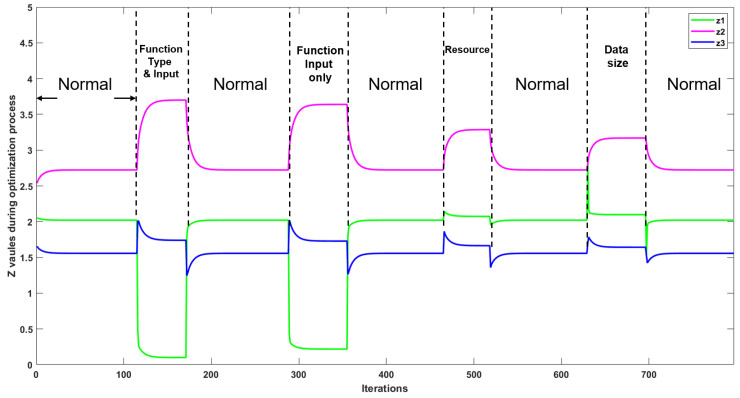
The *z* values of devices 1, 2, and 3 under different anomalies in the SS.

Note that anomalies can happen on any devices, and in this section we evaluate the anomaly detection based on anomalies occurring on device number one. This considers a reasonable scenario in a real-world IoT network, where a small number of devices (i.e., one device in our system) are attacked while the majority of devices (i.e., the other two devices) are maintained as normal. Figure 7 demonstrates the real-time change of parameter z when anomalies happen on device one. A decrease in the *z* of device one ($z_{1}$) is accompanied by an increase in the *z* of device two and three ($z_{2}$ and $z_{3}$) when the function type and function input are manipulated in SS.

### 5.4. Setup for LSTM-Based Networks

For the one-layer LSTM architecture, the settings include an input feature length of 10, resulting in an input size of 6 × 10, which consists of $\left[v_{i}^{1},\cdots ,v_{i}^{10};z_{i}^{1},\cdots ,z_{i}^{10}\right]$, where i=1,2,3 indicates the IoT device number. The step size is set as 5 and the hidden size of LSTM is set as 100. The bi-LSTM model is established based on this one-layer LSTM architecture with bidirectional mechanism. The stacked-LSTM is composed by stacking two one-layer LSTM architectures. For LSTM-attention, a multi-head attention mechanism with two heads follows the one-layer LSTM architecture. The number of input units of attention is set as 100, the same as the hidden size of LSTM. For LSTM-encoder, an encoder–decoder based on the one-layer LSTM is established and trained at the first stage. Then the encoder part is using for extracting the hidden feature for detection. The simulation dataset is split as follows: 60% for training, 20% for model validation, and 20% for simulation testing. Finally, the LSTM-based models are tested using the practical dataset. Experiments are repeated ten times for each anomaly type, and the mean and standard deviation of prediction accuracy are presented in Table 3 for the simulation test and practical prediction. We first investigate the performance of one-layer LSTM separately for each anomaly type and apply different variants of LSTM-based designs to comprehensively assess the general detection capability for all types of anomalies of our interest.

## 6. Experimental Results

### 6.1. Anomaly Detection on SS

In this section, SS anomalies are detected and the model performance is evaluated with respect to the different anomaly types. Firstly, when generating data (i.e., ADMM parameters z and v) from the SS, we investigate the scenario where only one specific type of anomaly (manipulation of function input alone) happens repeatedly. Here, we should note that different one-layer LSTM models are trained for different scenarios that only consist of a specific type of anomaly, with 60% of the data specified as the training set, 20% of the data for validation, and 20% of the data for testing.

As shown in Table 3, anomalies caused by manipulating the utility function input only are detected with an accuracy of 98.14%. Similarly, we investigated the detection performances for the other two anomaly types, “function type and input” and “data size”. Our results show that both anomalies can be detected with relatively high accuracy (99.82% and 93.91%) for manipulations of utility function type and input and transmission data size, respectively. These separated detection accuracies for specific manipulations reveal that the deep-learning-based approach is able to extract the individual pattern of each type of manipulation with very high accuracy.

Furthermore, when generating data (z and v) from the SS, we also investigated the scenario where three types of anomalies appear randomly (only one anomaly happens each time but can be any one of the different anomaly types). Here, only one LSTM model is trained for detecting different anomalies using data from the SS, with 60% of the data used as the training set, 20% of the data for validation, and 20% of the data for testing, which is consistent with the previous setups.

Both four-class detection (with labels 0, 1, 2, and 3 for situations including normality and the different anomalies, respectively) and two-class detection (here, normality and manipulation of system resources are labelled as 0, and other manipulations are labelled as 1) were investigated. The prediction accuracy was found to be 92.35% for four-class anomaly detection and 98.81% for two-class anomaly detection.

The rule-based detection in the SS is based on thresholds by identifying to which extent the *z* value is changed. Here, the threshold was assumed to be 1% of the optimal transmission frequencies of the IoT devices. Given this setting, Table 4 demonstrates the detection results obtained using this approach. Specifically, comparing with Table 3, the general (two-class) results show that the LSTM-based detection can easily outperform the rule-based detection method.

### 6.2. Anomaly Detection on RS

In order to better represent detection of anomalies in a real-world IoT environment, different types of anomalies are detected using the RS in this section. We recall that the LSTM model was trained based on the simulated data from the SS and is tested using the data from the RS in this setup.

**Figure 8:**
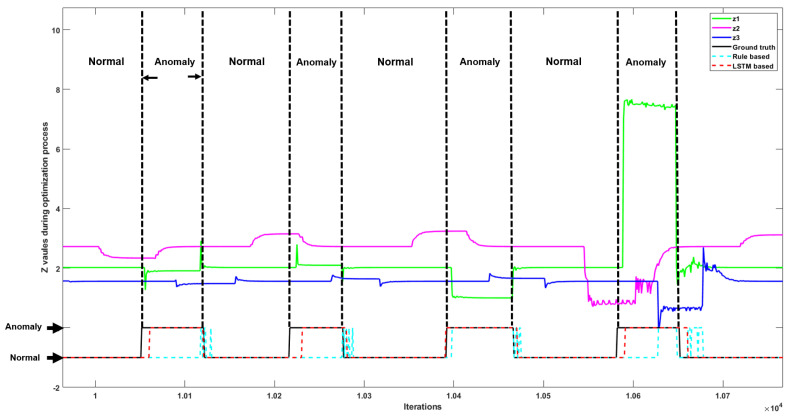
The *z* values of devices 1, 2, and 3 in the RS, including both normal and abnormal situations. General two-class detection results from the rule-based method (turquoise dotted line) and LSTM (red dotted line) are compared with the ground truth (black line).

**Figure 9:**
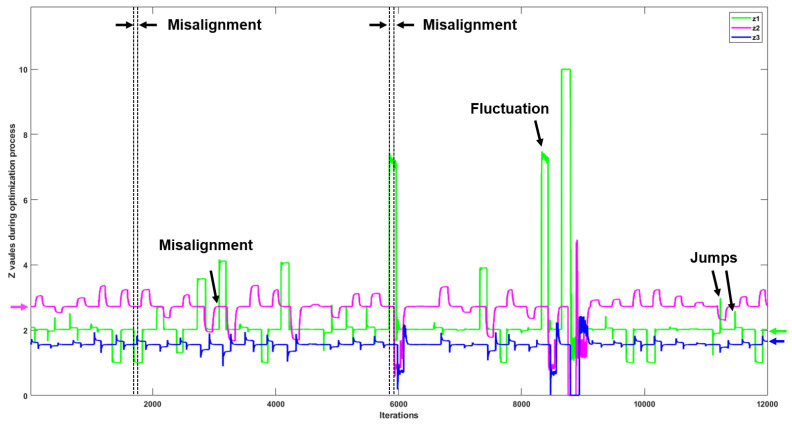
The *z* values of devices 1, 2, and 3 during the update process in the PS. The misalignments, fluctuations, and jumps can affect the performance of detection when detecting anomalies in real-world applications.

Our results in Figure 8 indicate the value of parameter *z* for devices 1, 2, and 3 (green, magenta, and blue lines, respectively) when the RS system is running normally, and in scenarios when three types of anomalies occur. In comparison to Figure 7, in this case, when an anomaly occurs, the value of parameter *z* for devices 1, 2, and 3 does not change at the same time, which causes the observed misalignments with respect to iterations of the ADMM algorithm. Figure 9 shows the variation of parameter *z* for devices 1, 2, and 3 on RS in a long timescale with misalignments, fluctuations, and jumps.

Comparing these results to those obtained for the SS experiments (Table 3), it is evident that the accuracy of anomaly detection for “function input only” from the RS (82.84%) is lower than that from the SS (98.14%) because of the misalignments between the *z* values of different devices. Similarly, detection of “function type and input” and “data size” anomalies in the RS (93.90% and 92.65%) had accuracies slightly lower than those presented in the SS simulation results. In addition, four-class detection and two-class detection were also investigated in the RS. As shown in Table 3, the general two-class detection achieved the highest accuracy of 96.28% in the RS, which indicates that the proposed LSTM-based method is promising for real-world IoT networks. However, with misalignments between parameter *z* for different devices, performances from the RS for four-class detection (i.e., an accuracy of 78.88%) and for two-class detection (i.e., an accuracy of 96.28%) are reduced compared to the performances from the SS (i.e., accuracies of 92.35% and 98.81% for four-class and two-class prediction, respectively).

Performance on the RS was poorer for the rule-based anomaly detection approach (Table 4), which may be due to the misalignments between parameter *z* between different devices. Since the rule-based approach leverages the simultaneous relationship between different transmission frequencies, it can be expected that a larger misalignment leads to a poorer performance for the rule-based approach. The general two-class detection results from rule-based and LSTM methods are compared against ground truth in Figure 8 in the RS. The detection results from the LSTM method better match the ground truth, while the rule-based method claims the anomalies incorrectly when there are misalignments and fluctuations in data flow.

**Figure 10:**
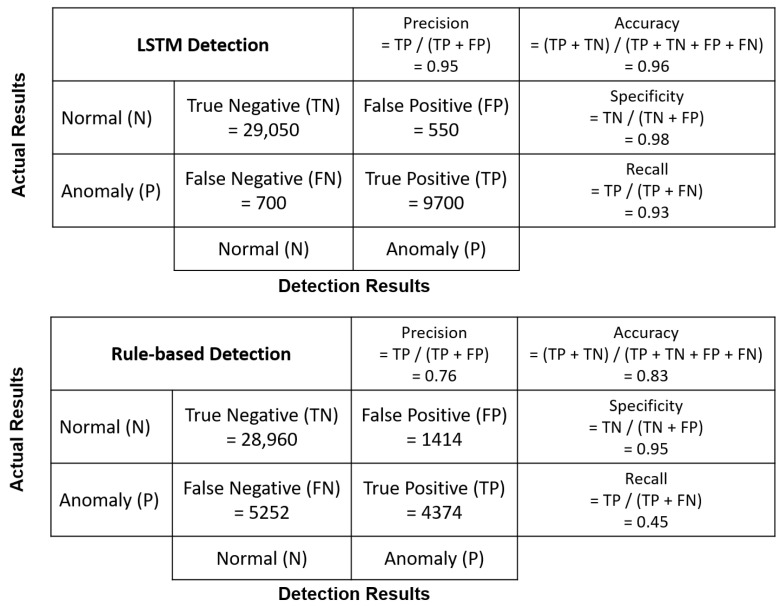
Confusion matrix for LSTM and rule-based detection on general two-class detection in the RS.

In order to provide more details for comparing the performance of rule-based and LSTM methods, precision, specificity, and recall metrics were calculated and are shown in Figure 10. Note that we calculate the metrics for LSTM every 10 time steps, as the input length of the LSTM model is taken as 10 in the model settings presented in Section 5, while the metrics for the rule-based method are computed by each time step. When the system is running normally, both methods have high specificity value (0.98 for the LSTM method and 0.95 for the rule-based method), which means that most of the time, both anomaly detectors will not raise alarm when the RS is running normally. However, the LSTM method obtains a higher recall value than the rule-based method for anomaly detection (0.93 for the LSTM method and 0.45 for the rule-based method), indicating that the LSTM method can raise alarm promptly when most malicious manipulations occur, but the rule-based method fails to detect most anomalies. Given the precision values (0.95 for the LSTM method and 0.76 for the rule-based method), the majority of anomalies identified by the LSTM method are real anomalies, and therefore the LSTM method is more acceptable for use in real-world applications.

## 7. Discussion

The results presented in Table 3 indicate that the one-layer LSTM detects anomalies more effectively in both the SS and the RS. In Table 3, the standard deviations of the detection results reveal that LSTM-based anomaly detection is robust, including the real-world system (RS). Although the accuracy decreases to 78.88% with some uncertainty (standard deviation of 3.80%) for four-class anomaly detection in the RS, the LSTM method can still obtain stable high performance (accuracy of 96.28% with standard deviation of 0.89%) for two-class anomaly detection. However, when detecting the “function input only” anomaly, the LSTM method has worse performance than the rule-based method. One possible reason for this is that the fluctuations and jumps in data flow shown in Figure 9 cause the uncertainty during the training process of LSTM models.

**Figure 11:**
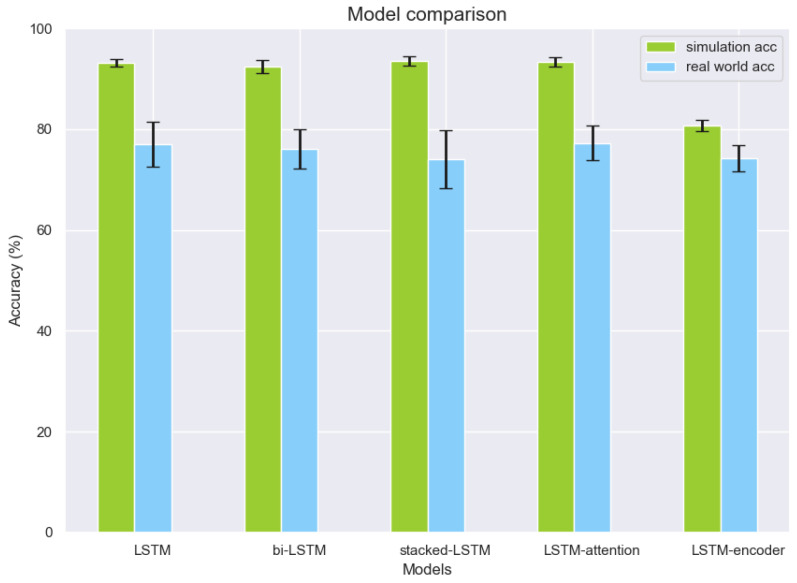
Four-class anomaly detection results from different deep learning architectures.

To further evaluate the general anomaly detection capabilities, one-layer LSTM, bi-LSTM, stacked-LSTM, LSTM-attention, and LSTM-encoder architecture are implemented and their results are reported for four-class anomaly detection. Figure 11 demonstrates the four-class detection accuracy for one-layer LSTM, bi-LSTM, stacked-LSTM, LSTM-attention, and LSTM-encoder. Each model is trained 10 times with different parameter initializations, and the average detection accuracy and standard deviation are calculated. Interestingly, we found that applying extra deep learning layers may not significantly improve the detection accuracy for the problem in the real-world environment. In particular, LSTM-attention-based architecture achieves slightly better results in comparison to the one using the basic LSTM architecture (i.e., comparable mean value but with fewer variances); however, both stacked-LSTM and LSTM-encoder-based architectures further degrade the performance accuracy in anomaly detection while increasing the model complexity as reported in Table 5. Additionally, in the table, bi-LSTM results in the longest inference time in both simulation and real-world environments. The shortest inference time is shown by LSTM-encoder, while it has the lowest detection performance accuracy in the group. With the comparable inference time consumption to LSTM-attention, one-layer LSTM has the minimum number of parameters to train, which means that this architecture can detect anomalies effectively with less computational resource for model training, albeit having a bit lower detection accuracy when compared to the best result achieved using the LSTM-attention-based method.

Table 4 indicates the detection results from the rule-based model. Detection of anomaly “data size” using the rule-based method has the lowest accuracy when compared to the other types of anomalies. The reason for this is that a change of transmission frequency for the manipulated device may lead to identical-trend changes of transmission frequencies for other devices, which prevents the rule-based detection working effectively. Interestingly, the detection accuracy for “data size” in the RS is very comparable to that in the SS, which may be largely caused by the misalignments and fluctuations in the RS data flow. However, as expected, the detection accuracies for other types of anomalies in the SS are greater than those in the RS. Finally, we also investigated the impact of thresholds in rule-based anomaly detection (Table 6). We note that the threshold plays an important role in anomaly detection. However, there is a trade-off in choosing the optimal threshold value for anomaly detection of the system. One the one hand, a network disturbance can easily trigger the anomaly in the network incorrectly if a small threshold applies. On the other hand, an anomaly may be ignored if the threshold is chosen too large. Therefore, a problem arises as to how to select an optimal threshold for anomaly detection in a practical IoT application (i.e., trial and error), which is another drawback of the rule-based method compared to the LSTM-based approach.

## 8. Conclusions

In this paper, we discussed anomaly detection in different scenarios, using both a mathematical-rule-based and LSTM-based approach, in a decentralised transmission frequency management system. IoT edge devices may suffer attacks that manipulate their transmission frequency and transmit data streams with an incorrect cadence. Considering that there are different manipulations that can change the transmission frequency, the rule-based approach demonstrates the internal process during an anomaly event but cannot reliably detect the anomaly in a practical environment. In contrast, the LSTM-based approach indicates greater potential for implementation in both simulations and real-world environments for the detection of abnormal transmission frequency.

In future work, we plan to extend the work through the following approaches: other data-driven algorithms (e.g., graph neural network [38,39]) for abnormal frequency detection will be investigated and a performance comparison undertaken; different topologies (e.g., partially connected topology [40]) will be applied to the IoT network to model the connection relationship between devices and the gateway; anomaly detection for transmission frequency manipulation of multi-devices will be investigated, focusing on the case where the majority of devices are manipulated; anomaly detection will be investigated in the case when a device suffers attacks by multiple manipulations at the same time; anomaly detection with plug-and-play capacity will be developed for application in anomaly detection when there are new devices connecting to or disconnecting from the gateway.

## Figures and Tables

**Table 1:** Utility functions.

Index	Utility Functions
1	$f_{1}\left(x_{1}\right)={\left(x_{1}-9\right)}^{2}+x_{1}^{3}$
2	$f_{2}\left(x_{2}\right)={\left(x_{2}-4\right)}^{2}$
3	$f_{3}\left(x_{3}\right)={\left(2x_{3}-6\right)}^{2}+x_{3}^{3}$

**Table 2:** Utility functions set.

Utility Functions
$f_{j}\left(x_{j}\right)={\left(x_{j}-9\right)}^{2}+x_{j}^{3}$
$f_{j}^{*}\left(x_{j}\right)=\exp\left(x_{j}-9\right)$
$f_{j}^{*}\left(x_{j}\right)=1/\left(x_{j}-9\right)$
$f_{j}^{*}\left(x_{j}\right)=\log\left(1+\exp\left(x_{j}-9\right)\right)$

**Table 3:** Accuracy of LSTM-based anomaly detection.

Anomaly Types	Simulation	Real-World System
Function input only	98.14% ± 0.52%	82.84% ± 3.81%
Function type and input	99.82% ± 0.01%	93.90% ± 1.52%
Data size	93.91% ± 1.00%	92.65% ± 0.85%
General (two-class)	98.81% ± 0.38%	96.28% ± 0.89%
General (four-class)	92.35% ± 0.84%	78.88% ± 3.80%

**Table 4:** Accuracy of rule-based anomaly detection.

Anomaly Types	Simulation	Real-World System
Function input only	97.48%	86.53%
Function type and input	99.65%	80.21%
Data size	65.26%	66.59%
General (two-class)	91.78%	83.34%

**Table 5:** The complexity comparison of different architectures.

Complexity	LSTM	bi-LSTM	Stacked-LSTM	LSTM-att.	LSTM-en.
Num. of model parameters	43,204	86,404	123,604	73,204	124,210
Simulation inference (s)	0.66	1.05	0.93	0.56	0.36
Real-world inference (s)	0.60	1.13	0.93	0.52	0.33

**Table 6:** Accuracy of rule-based anomaly detection for utility function input under different thresholds.

Thresholds	Real-World System
1% optimal frequency	86.53%
5% optimal frequency	87.90%
10% optimal frequency	87.02%
15% optimal frequency	86.62%
30% optimal frequency	83.23%
50% optimal frequency	77.10%

## Data Availability

Not applicable.

## Abbreviations

The following abbreviations are used in this manuscript:

MWF	Maximum writing frequency
DFWF	Data flow writing frequency
SS	Simulation system
RS	Real-world system
